# Solid phase thermal conductivity model of alpine meadow soils based on fractal theory

**DOI:** 10.1038/s41598-025-91128-3

**Published:** 2025-02-21

**Authors:** Wanjun Huang, Xuesong Mao, Qian Wu

**Affiliations:** 1 Civil Engineering and Transportation Academy, Qinghai Minzu University, Xining, 810007 People’s Republic of China; 2 Highway Academy, Changan University, Xian, 7100000 People’s Republic of China

**Keywords:** Model, Thermal conductivity, Fractal theory, Alpine meadow, Roots, Civil engineering, Computational methods

## Abstract

The determination of thermophysical parameters is crucial in the hydro-thermal coupling calculations of permafrost. Alpine meadows can affect the heat exchange inside and outside the soil due to the well-developed root, which can have an impact on the stability of permafrost. At present, the research on the thermal conductivity of alpine meadows is less, and most of the research focuses on the thermal conductivity of rock and soil. In this paper, according to the distribution and development characteristics of roots in the soil, a theoretical model of thermal conductivity of root-soil in alpine meadow based on thermal resistance is established by using fractal theory. The reliability of the theoretical model was verified by introducing the established solid-phase thermal conductivity model into the geometric mean model. The effects of the ratio of root area to total area(RRATA), tortuosity fractal dimension, and area fractal dimension on thermal conductivity are analyzed. The model constructed in this paper provides a theoretical basis for further research on the physical model of thermal conductivity of meadow porous media.

## Introduction

As the core organ of plant development, the root plays an important role in water transport and nutrient transport. It not only affects the mechanical properties of soil^1–3^ but also affects the physical properties^4^. In the permafrost region of the Qinghai-Tibet Plateau, the dynamic change of soil water and heat is not only closely related to climate change but also closely related to the physical properties of the soil itself. Alpine meadow, as the main vegetation type of the Tibetan Plateau, is characterized by the slow development of the above-ground part and huge underground root storage. And the abundant root makes the meadow layer has low thermal conductivity, which can reduce the heat exchange above and below the meadow layer, and keep the perennial permafrost stable. Therefore, it is necessary to study the effect of the root on the thermal conductivity of meadow soil.

The thermal conductivity of the meadow is mainly influenced by the solid skeleton, moisture, and internal air. The solid skeleton includes not only the soil particles but also the crisscross roots in the soil. Since the thermal conductivity of roots is small, it has a great influence on the thermal conductivity of the meadow. However, it is difficult to directly observe and measure the root because it is deeply buried in the ground. The traditional measurement indexes such as root-to-crown ratio, dry weight, and fresh weight can reflect the root growth to a certain extent, but it is difficult to reflect the root branching and structural changes. Indicators such as root length density and root weight density can reflect the distribution and branching of the root system in the soil, but they are very difficult to measure, which is time-consuming and laborious^5^. In contrast, fractal theory can reflect the geometric morphological characteristics of the root system through fractal characteristic parameters, and it is time-saving and convenient^6^. Therefore, it is a feasible method to construct the thermal conductivity model of the meadow by using fractal theory.

The application of fractal theory in thermal conductivity is mostly in porous media. Shi et al.^7^ derived a generalized heat conduction equation in porous media based on fractal theory. Fan et al.^8^ established a fractal model to predict the effective thermal conductivity of wood by the thermal resistance method. Xia et al.^9^ established a fractal model for the thermal conductivity of insulating fibre and analyzed the effects of porosity, pore area fractal dimension, and tortuosity fractal dimension on their thermal conductivity. Yu et al.^10^ proposed a fractal model to calculate the effective thermal conductivity of wood cross grain by using the series-parallel model and thermal resistance theory. Kou et al.^11^ obtained a non-dimensional calculation mode for the effective thermal conductivity of saturated and unsaturated porous media based on the thermal-electrical analogy principle and statistical self-similarity method. Jin et al.^12^ constructed a fractal structure with three-phase states based on a multi-stage Sierpinski-carpet structure and derived an effective thermal conductivity model.

At present, the application of fractal theory in the thermal conductivity model mostly focuses on porous media structure. Meadow is a special porous medium, the difference between general rock and soil is that the solid phase includes roots. There are few studies on the thermal conductivity of meadows, and few scholars have used fractal theory to construct root-soil thermal conductivity models. In this paper, the heat conduction process of the solid phase of the meadow was analyzed. Taking the root morphological characteristics of alpine meadows as the entry point, a model of thermal conductivity of root-soil solid phase based on thermal resistance theory was constructed using fractal theory. The reliability of the calculated model was also verified with experimental data, and the influence of relevant parameters on the thermal conductivity of meadow soil was quantitatively analyzed and evaluated.

## Fractal structure of roots in the alpine meadow

### Test materials and methods

#### Materials

**Fig. 1:**
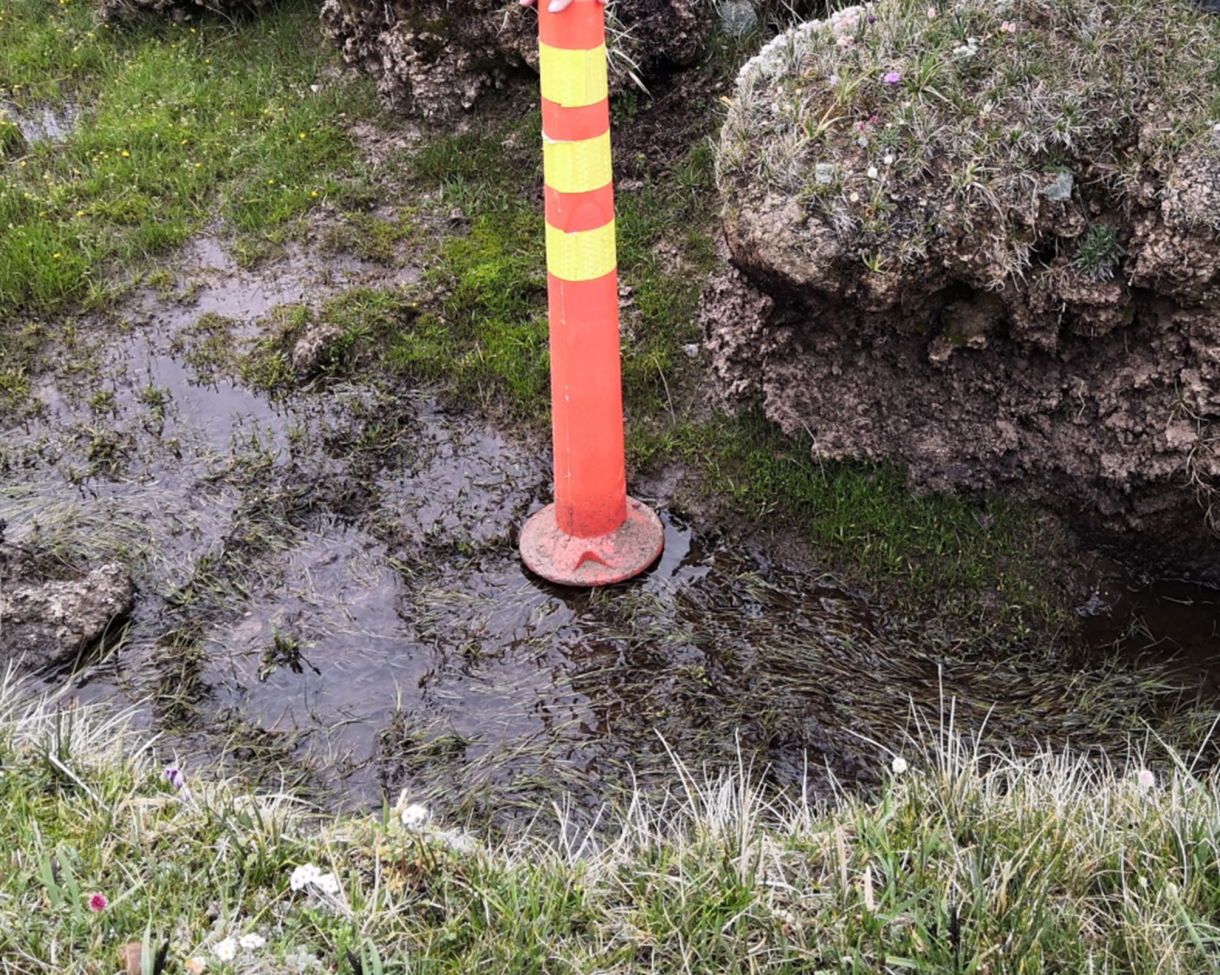
Alpine meadow.

The specimens were taken from a typical surface meadow layer in the plateau hinterland at an altitude of 4600–4700, as shown in Fig. 1. In order not to disturb the soil samples and for portability, the meadow soil was cut into 0.5*0.5*0.3 m^3^ rectangles. The samples were sealed with waterproof geotechnical material and marked with the soil sample number. The samples were then transported directly to the laboratory for refrigeration to prevent the plant fibres in the samples from being decomposed.

#### CT scanning test

**Fig. 2:**
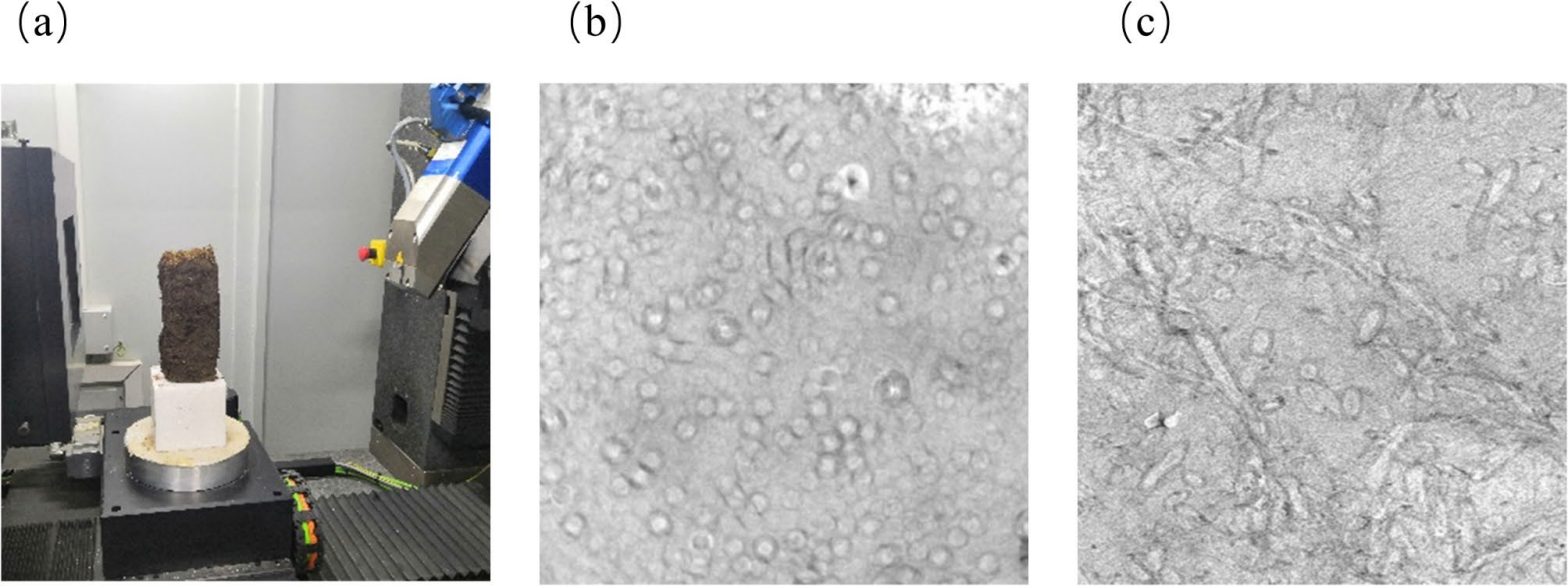
CT images of the meadow: (**a**) Specimen preparation for scanning; (**b**) cross-section; (**c**) longitudinal section.

The instrument used for the CT scanning test in this paper is the YTU225d X-ray CT system produced by Yxlon, Germany. To ensure the scanning accuracy and take into account the representativeness of soil samples, the sample was 25 cm in height, 10 cm in width, and 10 cm in length, as shown in Fig. 2(a). The CT images of the specimen in cross-section and longitudinal section were shown in Fig. 2 (b) and (c) respectively. From Fig. 2 (b), it can be found that roots of different diameters are scattered in the soil and the roots are perpendicular to the section. Whereas, from Fig. 2 (c), it can be found that the distribution of roots in the soil does not always follow the direction of heat flow.

### Acquisition and calculation of root characteristic parameters

#### Diameter of the root

**Fig. 3:**
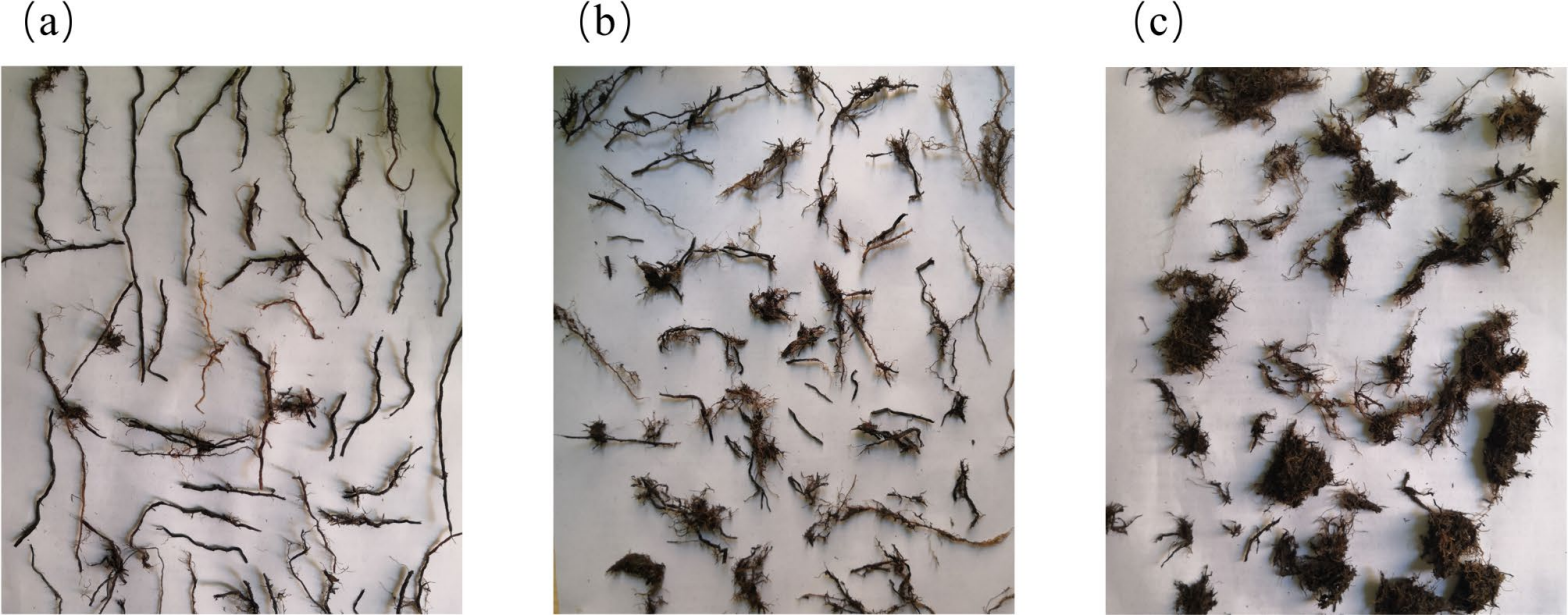
Root morphology: (**a**) Main root; (**b**) Secondary roots; (**c**) Capillary root.

The undisturbed soil was washed and flushed to obtain a complete root morphology, as shown in Fig. 3. Then the maximum diameter and minimum diameter of roots were measured by a vernier caliper, and the statistical results were shown in Table 1. According to the statistical results, the ratio of minimum diameter to maximum diameter is between 0.006 and 0.13.

**Table 1:** Statistical results of root diameter.

Root grades	Number of samples	Maximum value /mm	Minimum value /mm	Mean value /mm
Main root	60	5.31	0.42	2.16
Secondary root	212	2.06	0.12	0.95
Capillary root	154	0.23	0.03	0.15

#### Calculation of area fractal dimension of the root

**Fig. 4:**
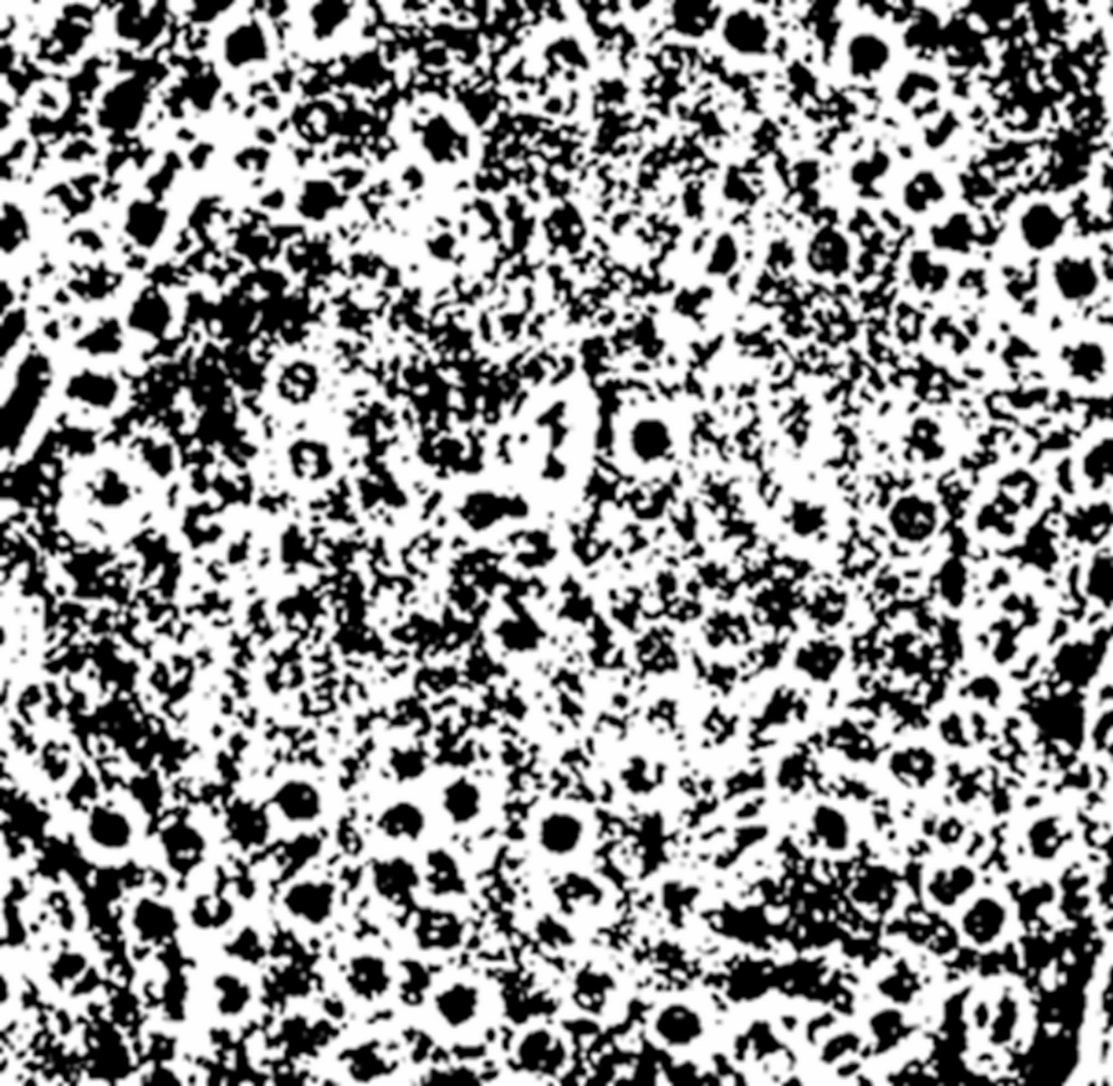
Binary graph of cross-section.

**Fig. 5:**
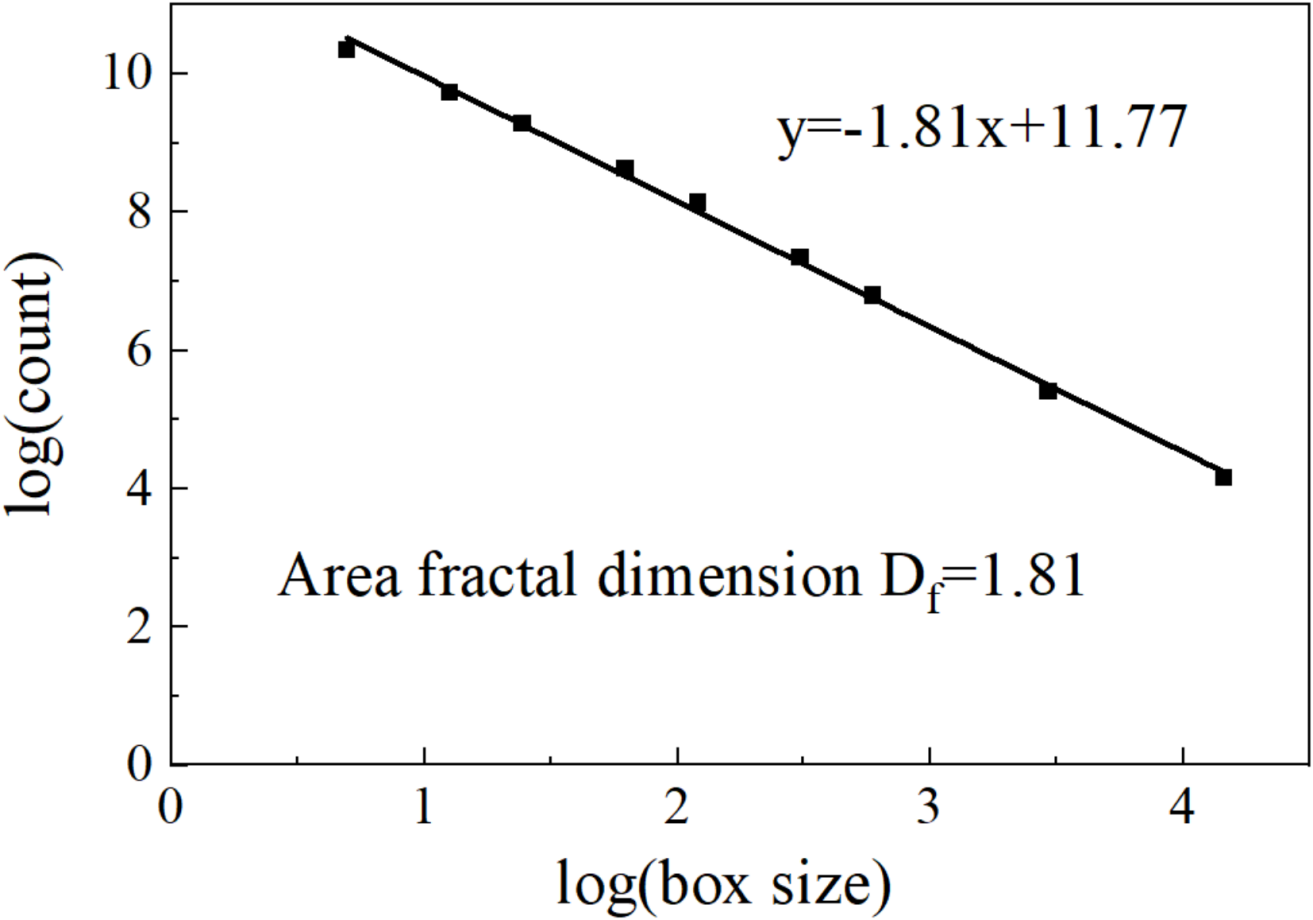
Fitting curve of root area fractal dimension.

First, the CT image of the cross-section (Fig. 2(b)) was binarized using image-j software, and the processed images are shown in Fig. 4. Then the image-j software was continued to be used to calculate the box dimensions of Fig. 4, and the logarithmic fit curve of the box dimensions and the number of boxes was shown in Fig. 5. It can be seen from Fig. 5 that the box dimension fitting curve of Fig. 4 has good linear characteristics, which proves the rationality of using the fractal method to study the thermal conductivity of alpine meadows. In Fig. 5, the slope of the fitting line is the required box dimension. Due to the characteristics of the box dimension itself and the possible errors in the measurement process, the results obtained can only be regarded as the estimation of the fractal dimension. According to the above steps, the box dimensions of the cross sections at depths of 5, 10, 15, and 20 cm are obtained, and the calculation results are shown in Table 2.

**Table 2:** Root area fractal dimension at different depths.

Depth/(cm)	${D}_{f}$
5	1.81
10	1.82
15	1.81
20	1.79

#### Calculation of tortuosity fractal dimension of the root

The tortuosity fractal dimension of roots represents the bending degree of roots in meadow soil. The greater the tortuosity fractal dimension, the higher the bending degree of roots. The roots of the meadow are generally formed by multiple roots such as the main root, secondary root, and capillary root. The diameter of the main root was the largest and longest, followed by the secondary root, and the capillary root was the smallest. Therefore, the fractal dimension of different levels of roots needs to be measured. The intact root morphology was obtained by washing the undisturbed soil and photographed by a camera. Then the roots at all levels were randomly extracted by image-j software, and the tortuosity fractal dimension of the streamlines of roots at all levels was calculated. The tortuosity fractal dimension was calculated by taking 10 streamlines for each level root, and then the average value was taken as the tortuosity fractal dimension for that root level. The calculation results of the different levels of roots are shown in Table 3.

**Table 3:** Tortuosity fractal dimension under different root grades.

Root grades	${D}_{t}$
Main root	1.60
Secondary root	1.57
Capillary root	1.55

## Theoretical model

The distribution of the root system in the soil is very complex and typical of a fractal structure. Therefore, it is very effective to apply fractal theory to study the root system of plants. Fractal geometry differs from Euclidean geometry in that its dimensions are not integers. Considering that the distribution of roots in the alpine meadow can be characterized by fractal geometry, this paper developed a predictive thermal conductivity model for alpine meadows based on previous research.

### The hypothesis of the model

A theoretical model for predicting the thermal conductivity of meadow soils has been established based on fractal theory, using the fundamental Fourier law of heat transfer and thermoelectric analogy analysis. Due to the complexity of the heat transfer mechanism, an accurate theoretical model is difficult, the following assumptions have been made to simplify the analysis: ①The diameter of the root system inside the meadow soil is constant along the direction of heat flow, but within the cross-section perpendicular to the direction of heat flow the diameter of the root system varies in a wide range, and the ratio of the minimum diameter to the maximum diameter is less than 0.01, i.e. min/max < 0.01^13^;②The root is assumed to be isotropic and the effect of internal porosity of the root is not considered; ③Thermal conductivity of soil particles and roots does not change with temperature under atmospheric pressure.

### Physical model of thermal conductivity

**Fig. 6:**
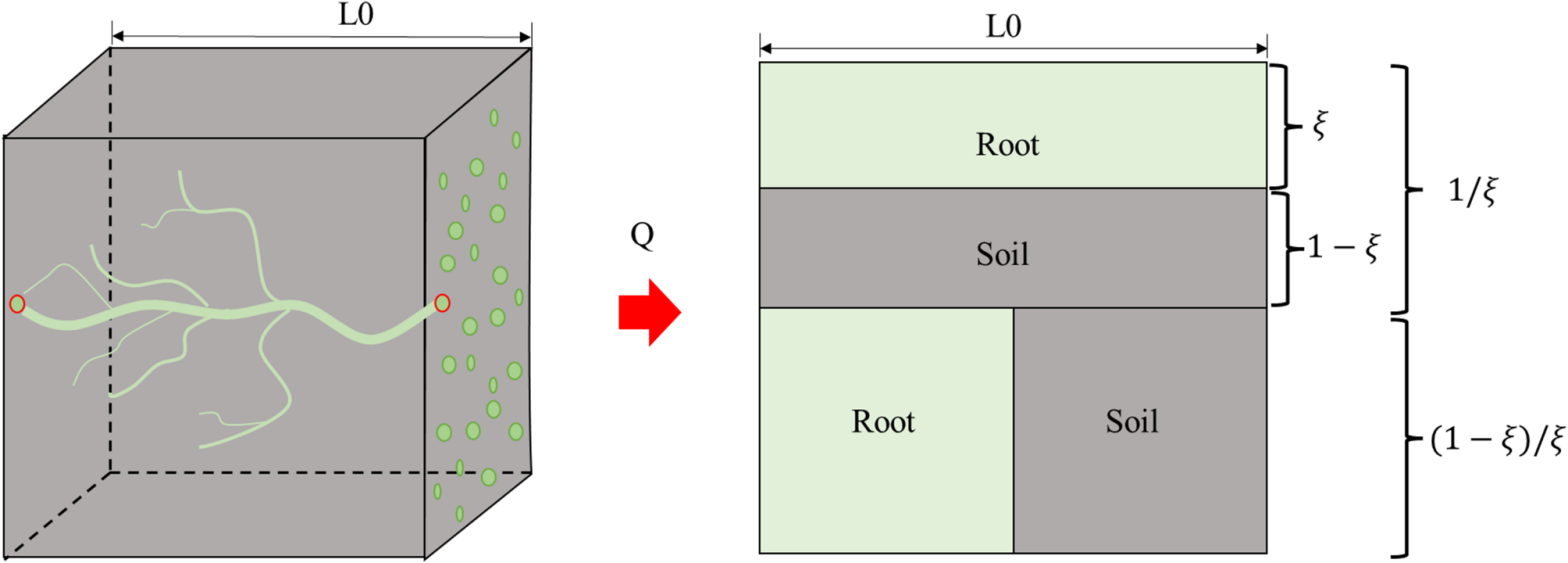
Schematic diagram of the equivalent thermal conductivity channel of the root-soil in series and parallel.

Figure 6 shows the physical model of the root-soil solid phase and the schematic diagram of root-soil series-parallel equivalent thermal conductivity channels. According to the spatial structure characteristics of the root in the soil, the root distribution in three-dimensional space is abstracted into two parts consisting of parallel with heat flow and vertical with heat flow. Two fractal dimensions are thus introduced to describe the microstructure of the roots: the root area fractal dimension (${D}_{f}$) relates the diameter of the root to the number of roots parallel to the heat flow direction, and the tortuosity fractal dimension (${D}_{t}$) of the root describes the length and bending degree of the heat flow channels.

### Mathematical model

#### Fractal theory of roots

The basic relation satisfied by the fractal geometry theory proposed by Mandelbrot^14^ is shown in Eq. (1).

1 $$N(\delta ) \propto {(\delta )^{{D_f}}}$$

where ${D_f}$ is the area fractal dimension; *δ* is scale of measurement; *N(δ)* is measurement results.

According to the basic defining equation of this theory, Mandelbrot considers that the number of islands on Earth and their area satisfy the following power law relationship:

2 $$N(r \geqslant {r_{\hbox{min} }})={({r_{\hbox{max} }}/{r_{\hbox{min} }})^{{D_f}}}$$

In the formula, $N$ is the number of islands, $r$ is the equivalent radius of islands, ${r_{\hbox{min} }}$ is the minimum equivalent radius of islands, and ${r_{\hbox{max} }}$ is the maximum equivalent radius of islands.

The fractal theory mentioned above is now widely used in porous media^15^. The distribution of roots also satisfies the fractal theory, so it is feasible to use Eq. (2) to calculate the number of roots in the meadow section. In addition, the number of roots in meadows is large and their metric scales such as area and radius (diameter) can be approximated as continuous and differentiable functions. Therefore, the number of roots with a radius in the range of $r$ and $r+dr$ can be obtained by differentiating Eq. (2) as follows.

3 $$- dN={D_f}{r_{\hbox{max} }}^{{{D_f}}}{d^{ - ({D_f}+1)}}dr$$

Equation (3) is the fractal scalar differential relationship between the number of roots and the radius of the root system in the interval from radius $r$ and $r+dr$. The negative sign indicates that the number of pores decreases with increasing radius.

The area fractal dimension of pores in porous media was derived as a function of porosity and pore diameter according to Koponen^16^ and Yu^17^. In this paper, the relationship between the area fractal dimension of roots and the RRATA and root diameter is constructed as shown in Eq. (4):

4 $${D_f}=2 - \ln (\zeta )/\ln ({r_{\hbox{min} }}/{r_{\hbox{max} }})$$

According to the scaling relationship of fractal theory, Wheatcraft et al.^18^ believed that when the fluid passes through porous media, the length of streamline and measurement scale also meet the fractal power law, as shown in Eq. (5):

5 $${L_t}(r)={r^{1 - {D_t}}}{L_0}^{{{D_t}}}$$

Where, ${L_t}$ is the actual streamline length; ${L_0}$is the straight-line distance; $r$is the measurement scale; ${D_t}$is the fractal dimension describing the Bending degree of streamline, 1<${D_t}$<2.

#### Thermal conductivity model based on fractal theory

Based on the arithmetic average combination of the two-phase solid-fluid component series-parallel model and the above assumptions, the fractal theoretical model for the thermal conductivity of the root-soil can first be expressed as Eq. (6):

6 $${\lambda _{effective,total}}=\frac{{{\lambda _{effective,parallel}}*{\lambda _{effective,series}}}}{{(1 - \psi ){\lambda _{effective,series}}+\psi {\lambda _{effective,parallel}}}}$$

Where${\lambda _{effective,total}}$is the thermal conductivity of meadow solid phase at macro scale; ${\lambda _{effective,parrallel}}$is the thermal conductivity contribution term corresponding to the parallel connection of components in the fractal model under the assumptions; ${\lambda _{effective,series}}$is the thermal conductivity contribution term corresponding to the series connection of components in the fractal model; $\varPsi$ is the proportion of channels perpendicular to the heat flow to the total channels.

1. $${\lambda _{effective,parrallel}}$$

**Fig. 7:**
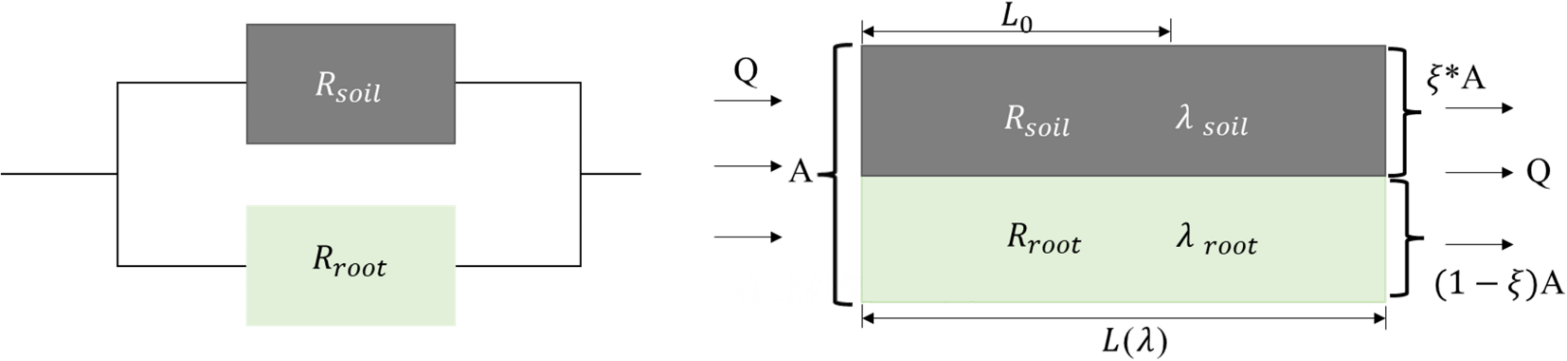
Schematic diagram of parallel thermal resistance of root-soil thermal conductivity.

The equivalent thermal resistance of the parallel section is shown in Fig. 7.

In Fig. 7, $Q$ is the heat flux, $\xi$ is the ratio of root area to total area (RRATA), and $A$ is the macro cross-sectional area of the meadow, then the area of heat transfer through the root is $\xi*A$, and the area of heat transfer through soil is (1-$\xi$)*$A$; ${\lambda}_{soil}$ and ${\lambda}_{root}$ are the thermal conductivity of soil and root, respectively; ${R}_{soil}$ and ${R}_{root}$ are the total thermal resistance of the heat conduction channels of soil and root, respectively; ${L}_{0}$ is the characteristic length of the root system, and $L$($\lambda$) is the length of the actual bending channel during heat conduction.

Because the solid phase is composed of roots and soil particles, the distribution of roots in the soil can be characterized by fractal geometry theory. According to Eq. (3), the root area on the cross-section of the meadow can be calculated, and the calculation results are as Eq. (7):

7 $${A_r}= - \int\limits_{{{r_{\hbox{min} }}}}^{{{r_{\hbox{max} }}}} {A(r)} dN=\frac{{\pi {D_f}}}{{(2 - {D_f})}}{r_{\hbox{max} }}^{2}\left[ {1 - {{({r_{\hbox{min} }}/{r_{\hbox{max} }})}^{(2 - {D_f})}}} \right]$$

Here, ${A_r}$ is the root area on the cross-section of the meadow.

Then the area of the whole section can be expressed as Eq. (8):

8 $$A=\frac{{{A_r}}}{\zeta }=\frac{{\pi {D_f}}}{{(2 - {D_f})\zeta }}{r_{\hbox{max} }}^{2}\left[ {1 - {{({r_{\hbox{min} }}/{r_{\hbox{max} }})}^{(2 - {D_f})}}} \right]$$

Then the thermal resistance of a single root system can be expressed as Eq. (9):

9 $$R(r)=\frac{{L(r)}}{{\pi {r^2}{\lambda _r}}}$$

Then the parallel thermal resistance of all roots with radius r is expressed as Eq. (10)

10 $${R_N}(r){\text{=}}\frac{{R(r)}}{{N(r)}}=\frac{{{L_0}^{{{D_t}}}{r^{{D_{\text{f}}} - {D_t}}}}}{{\pi {\lambda _r}{D_f}{r_{\hbox{max} }}^{{{D_f}}}}}$$

Thus, the total thermal resistance of the root is expressed as Eq. (11).

11 $${R_r}=\frac{1}{{\int\limits_{{{r_{\hbox{min} }}}}^{{{r_{\hbox{max} }}}} {\frac{1}{{{R_N}({\text{r}})}}} d{\text{r}}}}=\frac{{{L_0}^{{{D_t}}}({D_t} - {D_f}+1)}}{{\pi {\lambda _r}{D_f}{r_{\hbox{max} }}^{{{D_t}+1}}\left[ {1 - {{({r_{\hbox{min} }}/{r_{\hbox{max} }})}^{({D_t} - {D_f}+1)}}} \right]}}$$

The total thermal resistance of the soil particles is expressed in Eq. (12).

12 $${R_s}=\frac{{{L_0}}}{{(1 - \zeta )A{\lambda _s}}}$$

The total thermal resistance of the root system and soil particles is known, then according to the parallel relationship, the total parallel thermal resistance can be expressed as Eq. (13).

13 $${R_{parallel}}=\frac{1}{{\frac{1}{{{R_r}}}+\frac{1}{{{R_s}}}}}=\frac{{{L_0}}}{{A{\lambda _{effective,parallel}}}}$$

Substituting Eqs. (11) and (12) into Eq. (13), the thermal conductivity of the root-soil parallel part can be obtained as Eq. (14).

14 $${\lambda _{effective,parallel}}=\frac{{{L_0}}}{A}(\frac{1}{{{R_r}}}+\frac{1}{{{R_s}}})={\lambda _s}(1 - \zeta )+\frac{{\zeta {\lambda _r}(2 - {D_f}){r_{\hbox{max} }}^{{{D_t} - 1}}(1 - {{({r_{\hbox{min} }}/{r_{\hbox{max} }})}^{{D_t} - {D_f}+1}})}}{{{L_0}^{{{D_t} - 1}}({D_t} - {D_f}+1)(1 - {{({r_{\hbox{min} }}/{r_{\hbox{max} }})}^{(2 - {D_f})}})}}$$

(2) $${\lambda _{effective,series}}$$

**Fig. 8:**
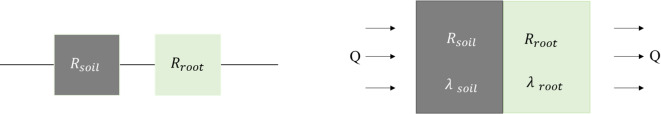
Diagram of series thermal resistance of thermal conductivity of root-soil.

The equivalent thermal resistance of the series part is shown in Fig. 8.

Since the heat flow direction is perpendicular to the root streamline direction in the one-dimensional steady-state heat conduction process, the area parameter of heat transfer direction is difficult to determine. Therefore, according to the basic theory of series-parallel thermal resistance, the effective thermal conductivity in series can be expressed as Eq. (15):

15 $${\lambda _{effective,series}}=\frac{1}{{\frac{\zeta }{{{\lambda _r}}}+\frac{{1 - \zeta }}{{{\lambda _s}}}}}{\text{=}}\frac{{{\lambda _r}{\lambda _s}}}{{\zeta {\lambda _s}{\text{+}}(1 - \zeta ){\lambda _r}}}$$

Substituting Eqs. (14) and (15) into Eq. (6), the thermal conductivity of the meadow solid phase can be obtained as Eq. (16):

16 $${\lambda _{effective,total}}=\frac{{{\lambda _s}(1 - \zeta )+\frac{{\zeta {\lambda _r}(2 - {D_f}){r_{\hbox{max} }}^{{{D_t} - 1}}(1 - {{({r_{\hbox{min} }}/{r_{\hbox{max} }})}^{{D_t} - {D_f}+1}})}}{{{L_0}^{{{D_t} - 1}}({D_t} - {D_f}+1)(1 - {{({r_{\hbox{min} }}/{r_{\hbox{max} }})}^{(2 - {D_f})}})}}{\text{+}}\frac{{{\lambda _r}{\lambda _s}}}{{\zeta {\lambda _s}{\text{+}}(1 - \zeta ){\lambda _r}}}}}{{(1+\psi )\frac{{{\lambda _r}{\lambda _s}}}{{\zeta {\lambda _s}{\text{+}}(1 - \zeta ){\lambda _r}}}+\psi \left[ {{\lambda _s}(1 - \zeta )+\frac{{\zeta {\lambda _r}(2 - {D_f}){r^{{D_t} - 1}}(1 - {{({r_{\hbox{min} }}/{r_{\hbox{max} }})}^{{D_t} - {D_f}+1}})}}{{{L_0}^{{{D_t} - 1}}({D_t} - {D_f}+1)(1 - {{({r_{\hbox{min} }}/{r_{\hbox{max} }})}^{(2 - {D_f})}})}}} \right]}}$$

### Model verification

Because the model constructed above is a root-soil solid-phase model, it only reflects the effect of roots on the solid-phase thermal conductivity and does not reflect the situation in practice. Therefore, to be able to verify the experimental test values, it is also necessary to consider the effect of moisture and air on the thermal conductivity. The geometrical average model is widely used because of its high accuracy. The constructed solid-phase thermal conductivity model is introduced into the geometrical average model, and its expression is shown in Eq. (17):

17 $$\lambda = \lambda _{{_{{effective,total}} }}^{{1 - \phi }} \lambda _{w}^{{\phi S_{r} }} \lambda _{a}^{{\left( {1 - S_{r} } \right)\phi }}$$

Where $\lambda$ is the actual thermal conductivity considering the pore, $\phi$ is the porosity of the meadow, ${S_r}$ is the saturation, ${\lambda _{\text{a}}}$ is the thermal conductivity of air and takes the value of 0.6 W/(m·K), and ${\lambda _w}$ is the thermal conductivity of water and takes the value of 0.023 W/(m·K). The thermal conductivity of the solid phase (${\lambda _{_{{effective,total}}}}$) is calculated from Eq. (15), where the thermal conductivity of the soil particles is 2.5 W/(m·K), and the thermal conductivity of the roots is 0.086 W/(m·K).

The thermal conductivity of the meadow at different RRATA was tested by the plate method in the indoor experiment, and the physical parameters of the meadow at the time of the test are shown in Table 4.

**Table 4:** Data obtained from laboratory test.

RRATA /1	${d}_{max}$ /mm	${d}_{min}$ /mm	Water content/1	Porosity /1	Saturation /1	Thermal conductivity/ W/(m·K)
0	/	/	0.3	0.57	0.58	0.5190
0.05	5	0.05	0.3	0.58	0.53	0.4456
0.15	5	0.05	0.3	0.61	0.48	0.4099
0.25	5	0.05	0.3	0.67	0.42	0.3966

**Fig. 9:**
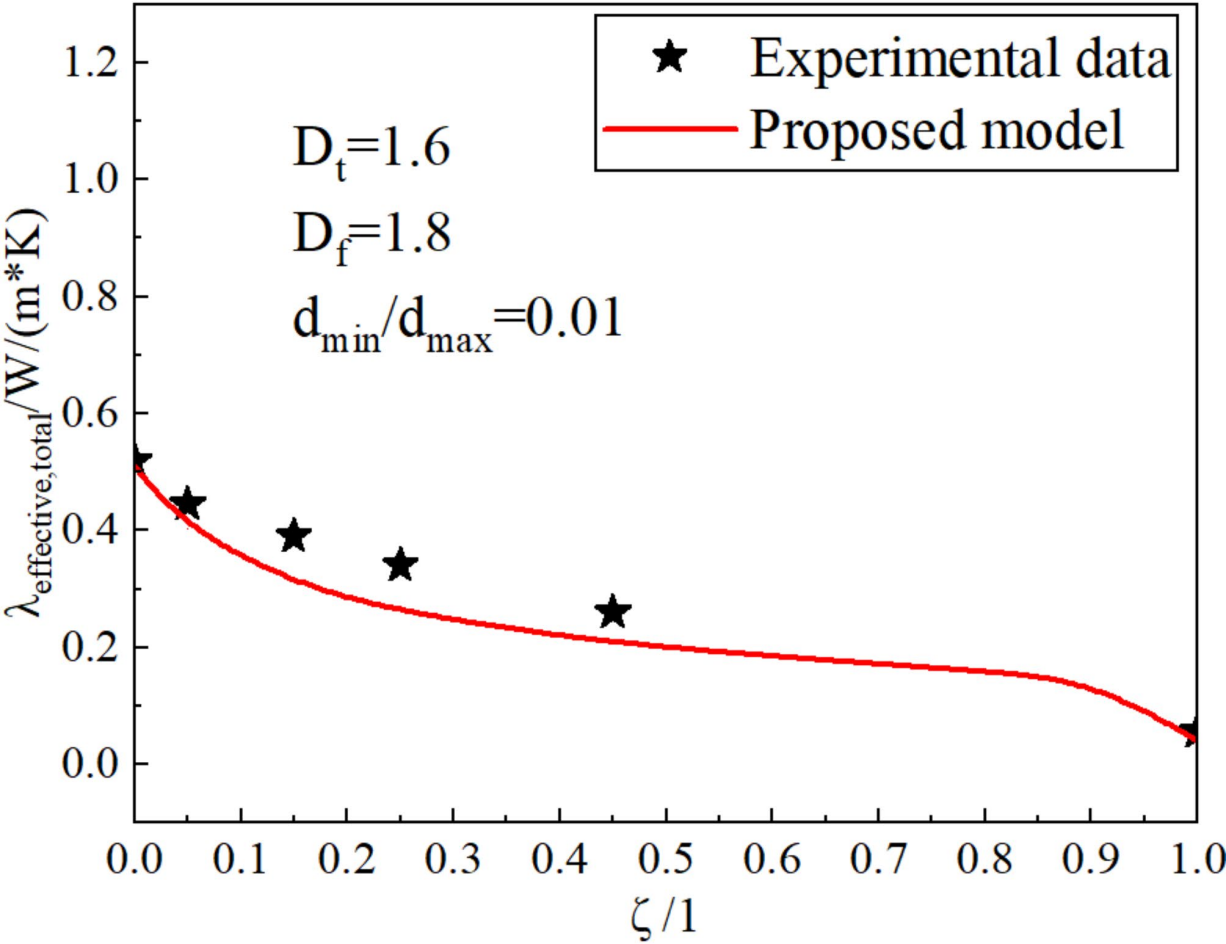
Comparison of experimental and theoretical values of thermal conductivity in the alpine meadow.

The experimental values at different RRATA were compared with the theoretical values, as shown in Fig. 9. It can be seen from the figure that the theoretical value is consistent with the experimental value, but the theoretical value is smaller than the experimental value. The reason for this phenomenon is that the influence of the micro-pores of the roots on the thermal conductivity is not considered when constructing the solid-phase thermal conductivity model of meadow soil. The thermal conductivity of roots in dry and wet conditions is quite different. The thermal conductivity of air is 0.023, while the thermal conductivity of water is 0.6, and the latter is 26 times that of the former. Because the root is assumed to be homogeneous and isotropic, the water content obtained from the indoor test is considered to exist only in the pores between the solid particles. Because the root is assumed to be a homogeneous material, the thermal conductivity of the root system does not change with the moisture content. However, due to the presence of water, the actual value of the thermal conductivity of the root is greater than the test value of the thermal conductivity of the root, so the predicted value calculated by the solid phase thermal conductivity model is always less than the actual value. This is the reason why the thermal conductivity model has an underestimation effect. Although the predicted value is smaller than the actual value, the RMSE is 0.054 W/(m·K), indicating that the model has reasonable acceptable accuracy and can be used to estimate the effective thermal conductivity of meadow soil under actual positive temperature conditions.

### Factors affecting root-soil thermal conductivity of alpine meadow

In this paper, a thermal conductivity model considering the spatial distribution of roots in soil is established. In this model, the RRATA, the tortuosity fractal dimension, and the area fractal dimension will affect the thermal conductivity. Therefore, the effect of the factors on the thermal conductivity is analyzed and discussed respectively.

#### Effect of the RRATA on thermal conductivity

**Fig. 10:**
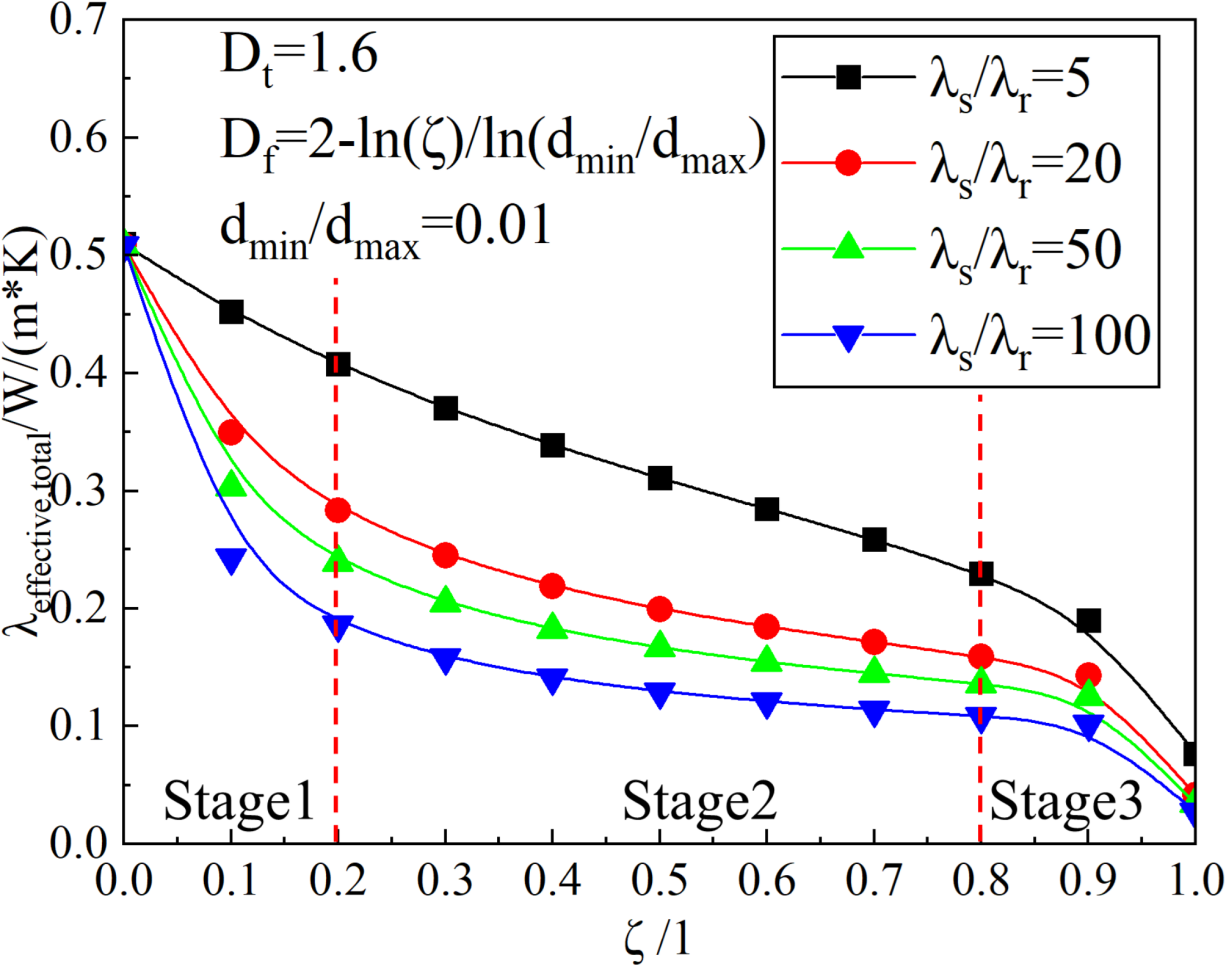
The change of thermal conductivity with RRATA.

The relationship between the thermal conductivity and the RRATA for different soil-root thermal conductivity ratios is given in Fig. 10. It can be seen from the figure that the thermal conductivity of alpine meadows decreases with increasing RRATA. The thermal conductivity of the soil skeleton is greater than that of the root system, and as the RRATA increases, the percentage of the soil skeleton decreases accordingly and the thermal conductivity decreases. The variation rate of thermal conductivity of alpine meadow can be divided into three intervals over the entire range of RRATA: when ξ < 0.2 (Stage 1), it is a high variation rate region, and the thermal conductivity of this interval decreases sharply; When 0.2 < ξ < 0 .08 (Stage) is the medium rate of change zone, the rate of change of thermal conductivity in this zone tends to be stable; While when ξ < 0 .08 (Stage 3) , it enters the high rate of change zone again. The thermal conductivity curve varies in an “S” pattern and this trend becomes more obvious as the soil-root thermal conductivity increases. The appearance of the above phenomenon is related to the variation in the thermal conductivity of the root. When the RRATA is 0.1 ~ 0.2, the greater the soil-root thermal conductivity ratio, the more sensitive the thermal conductivity to the root content. When RRATA is 0.1 ~ 0.2, the root content increases the thermal resistance of the soil. Therefore, the greater the soil-root thermal conductivity ratio, the greater the variation rate thermal conductivity of the alpine meadow. When the RRATA is 0.2 ~ 0.8, the change of thermal resistance is not sensitive to the change of root content, so the change rate of thermal conductivity tends to be flat. With the RRATA greater than 0.8, the connection between soil particles in the meadow is no longer continuous, which makes the thermal resistance grow rapidly, so the thermal conductivity decreases rapidly again. From the figure, we can see that the RRATA of 0.2 is a threshold value for the change of thermal conductivity, when RRATA is less than 0.2, the thermal conductivity is mainly dominated by the thermal conductivity of the soil skeleton, while when RRATA is greater than 0.2, the thermal conductivity is dominated by the root system. When RRATA is less than 0.8, the heat flow is mainly transferred through the soil skeleton, and with the increase of root content the thermal resistance increases rapidly making the heat flow decrease and the thermal conductivity decreases, and this stage of the thermal conductivity is more sensitive to the change of root content. When RRATA is 0.2 ~ 0.8, because the root system in the soil is more developed, the effect of thermal resistance on heat flow transfer due to the increase of root content has been weakened. Although the thermal conductivity decreases with the increase of root content, the magnitude decreases significantly, and this phenomenon is more obvious when the ratio of the thermal conductivity of the soil skeleton to the thermal conductivity of the root system is larger. With RRATA greater than 0.8, the meadow soil particles were no longer continuous both in the direction of heat flow transfer and perpendicular to it, so the thermal resistance grew rapidly and the thermal conductivity again decreased rapidly.

#### Effect of tortuosity fractal dimension of the root

**Fig. 11:**
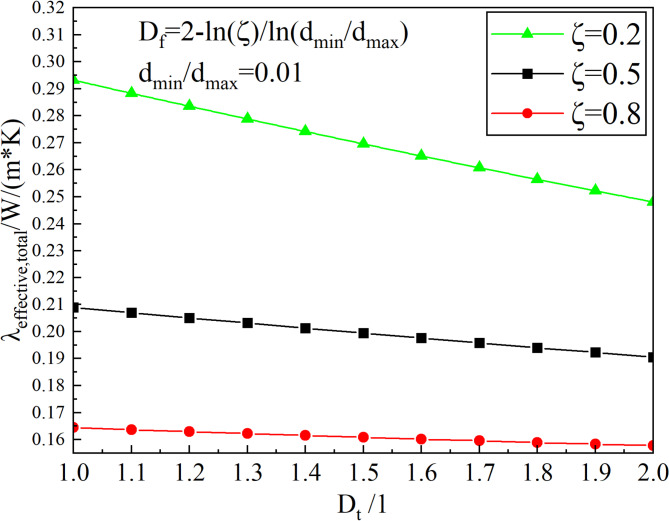
Effect of tortuosity fractal dimension on thermal conductivity.

The tortuosity fractal dimension reflects the bending degree of roots in the meadow and affects the path and distribution of heat flow in the heat conduction process. Figure 11 shows the relationship between the thermal conductivity of the alpine meadow and the tortuosity fractal dimension at different RRATA. As can be seen from the figure, the thermal conductivity decreases as the tortuosity fractal dimension increases. The larger the tortuosity fractal dimension, the more the channel is twisted and the longer the path through which the heat flow passes. When the tortuosity fractal dimension increases, the distance of heat transfer through the root system increases, i.e., the thermal resistance to heat conduction increases and the thermal conductivity decreases. However, the variation rate of the thermal conductivity decreases as the RRATA increases. When the RRATA is 0.2, the thermal conductivity decreases from 0.289 W/(m·K) to 0.252 W/(m·K) as the tortuosity fractal dimension increases from 1.1 to 1.9, which decreases by about 12.5%. When the RRATA is 0.8, the thermal conductivity only decreases by 0.005 W/(m·K) as the tortuosity fractal dimension increases from 1.1 to 1.9, which decreases by less than 4%. It can be seen that the effect of the tortuosity fractal dimension on the thermal conductivity is also closely related to the RRATA. When RRATA is less than 0.5, most of the medium in the meadow is soil particles, so the tortuosity of the root has a significant impact on the thermal resistance of the meadow and the variation rate of thermal conductivity is larger with the increase of RRATA. When RRATA is greater than 0.5, the total thermal conductivity is smaller because of the larger root content. At this point, the effect of the tortuosity fractal dimension on the thermal conductivity is no longer significant.

#### Effect of area fractal dimension of the root

**Fig. 12:**
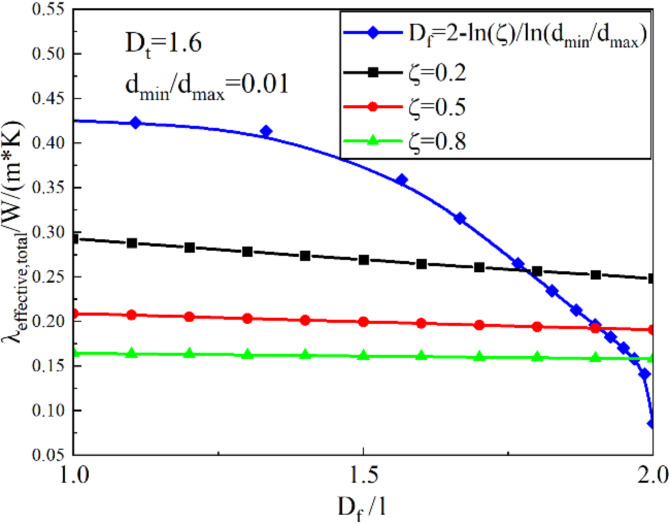
Effect of area fractal dimension on thermal conductivity.

The area fractal dimension is an important parameter used by fractal theory to describe the spatial irregularity and disorder of the root, which reflects the complexity of the root distribution. Figure 12 shows the relationship between the area fractal dimension and the thermal conductivity. The increase in the fractal dimension of the area indicates an increase in the root percentage, which will increase the thermal resistance and make the solid-phase thermal conductivity of the meadow soil decrease. In addition, when using the area fractal dimension as the independent variable, the thermal conductivity decreases as the area fractal dimension increases. The change rule is the same as the effect of tortuosity fractal dimension on thermal conductivity. It can be found that the effect of area fractal dimension as an independent variable on thermal conductivity is weak, especially when the RRATA is more than 0.5. When RRATA exceeds 0.5, the roots in the soil are developed, and the thermal conductivity is smaller than 0.21 W/(m·K). At this time, the increase of the area fractal dimension has no significant effect on the heat flow transfer. When the area fractal dimension is used as a non-independent variable, the thermal conductivity decreases from 0.423 W/(m·K) to 0.196 W/(m·K) as the area fractal dimension increases from 1.1 to 1.9, which decreases by 53.66%. It can be found that the variation in thermal conductivity is more obvious with the area fractional dimension as a non-dependent variable than with the area fractional dimension as an independent variable. The area fractal dimension reflects the proportion of roots on the cross-section. The larger the area fractal dimension is, the larger the proportion of roots is, and vice versa. With an area fractal dimension of 1, the predicted value tends to be close to the thermal conductivity of unsaturated soils, while with an area fractal dimension tending to 2, the predicted value is in between the thermal conductivity of roots and air. This variation in thermal conductivity when the area fractal dimension is used as a non-independent variable is closer to the actual situation. The above phenomenon is almost difficult to reflect when the area fractal dimension is an independent variable, and the higher the RRATA, the weaker the influence of the area fractal dimension on the thermal conductivity. Obviously, the area fractal dimension as an independent variable can not reflect the influence of the root system on the thermal conductivity. Only when it is used as a function of the RRATA, it is closer to reality. Therefore, it is reasonable and necessary to take the area fractal dimension as the function of RRATA and root diameter.

## Discussion

From the comparison between the predicted value and the measured value, the root-soil solid phase model can meet the engineering needs and can be used to estimate the thermal conductivity of meadow soil at a positive temperature. However, it should be noted that the model assumes that the root is a homogeneous, isotropic material, which is not consistent with the actual situation. From a microscopic point of view, a large number of micro-pores containing gas or water are distributed inside the roots, which will significantly affect the thermal conductivity of the roots. In addition, the root-soil solid phase thermal conductivity model only considers the heat transfer process of solid phase heat conduction in meadow soil and does not consider the influence of the gas phase and liquid phase on the thermal conductivity in meadow soil. Therefore, In the follow-up work, the solid-phase thermal conductivity model will be further optimized to improve its accuracy, and the effect of micro-pores on the thermal conductivity of the root will be analyzed. Then, on the basis of the optimized solid phase model, the pore structure and size characteristics of meadow soil will be further considered, and the thermal conductivity model of meadow soil including the solid phase, gas phase, and liquid phase will be constructed from the macro and micro perspectives. Finally, according to the actual working conditions of alpine meadow soil, based on fractal theory, thermoelectric analogy principle, and fractal-capillary theory, the thermal conductivity model of alpine meadow soil considering phase change will be constructed.

## Conclusion

1. In this paper, the basic principles of fractal geometry are applied to establish a solid-phase fractal thermal conductivity model for plateau meadows. The fractal expression for the effective thermal conductivity of the solid phase of the meadow is derived, and the parameters affecting the thermal conductivity of the solid phase of the meadow are obtained.
2. To verify the validity of the solid phase thermal conductivity, the constructed solid phase model was introduced into the geometric mean model, and the calculated theoretical value and experimental value were compared. The calculated RMSE and NRMSE of thermal conductivity of the present model are 0.054 W/(m·K) and 0.12 respectively, which indicate that the model has reasonable and acceptable accuracy.
3. The effects of RRATA, tortuosity fractal dimension, and area fractal dimension of the root on the thermal conductivity were systematically analyzed. The results show that: the thermal conductivity decreases with the increase of root proportion. When the RRATA is constant, the larger the tortuosity fractal dimension is, the smaller the thermal conductivity is. When the RRATA is greater than 0.5, the influence of the tortuosity fractal dimension on the thermal conductivity decreases. The area fractal dimension has a smaller effect on the thermal conductivity when used as an independent variable. When used as a non-independent variable, the area fractal dimension is a function of the root diameter and the root percentage, and the effect on the thermal conductivity is much closer to the actual situation. Therefore the area fractal dimension should be used as a non-independent variable.

## Data Availability

Some or all data, models, or codes that support the findings of this study are available from the corresponding author upon reasonable request (list items).

## Declarations

### Statement on the permission to collect plant root materials

This study hereby issued an official statement on its right to collect plant root materials for scientific research purposes. This activity is carried out in accordance with relevant laws and regulations to ensure the rational use and protection of plant resources. During the collection process, we will strictly adhere to national environmental protection and biodiversity conservation laws and regulations to prevent any adverse impact on the ecosystem. At the same time, necessary safety measures will be taken to avoid any potential negative effects on human health or animal welfare.We commit to making all collection activities transparent and open to public scrutiny. Should you have any questions or require further information, please do not hesitate to contact us.This statement aims to clarify our rights and obligations to ensure the legality and legitimacy of all relevant activities.
